# Mix-Proportion Design and Properties of Steel Slag-Based Composite Cementitious Materials Using a Constrained Four-Component Mixture Design

**DOI:** 10.3390/ma19204294

**Published:** 2026-10-12

**Authors:** Qiaoming Guo, Yuanbao Liu, Hui Chen, Zhengbin Luo, Bin Liu, Minghui Lin, Liu Yang, Zuming Chen, Yi Zhao

**Affiliations:** 1 Jiangxi Provincial Communications Investment Group Co., Ltd., Nanchang 330108, China; 2 Jiangxi JPCI Maintenance Technology Group Co., Ltd., Nanchang 330108, China; 3 School of Civil Engineering, Chongqing Jiaotong University, No. 66 Xuefu Road, Nan’an District, Chongqing 400074, China; 4 School of Materials Science and Engineering, Chongqing Jiaotong University, No. 66 Xuefu Road, Nan’an District, Chongqing 400074, China

**Keywords:** constrained mixture design, cementitious materials, mixture model, hydration characteristics, microstructure

## Abstract

**Highlights:**

• Seventeen SS-BFS-OPC-DG formulations were screened using a constrained four-component mixture design.
• A 7 d compressive-strength mixture model was established and evaluated statistically.
• Strength and hydration evolution from 3 d to 28 d were interpreted using XRD, SEM, TG, and FTIR evidence.

**Abstract:**

High-volume utilization of steel slag in cementitious materials requires a proportioning method that accounts for coupled effects among multiple components. This study investigated a four-component system comprising steel slag (SS), blast furnace slag (BFS), ordinary Portland cement (OPC), and desulfurized gypsum (DG), using 17 formulations within a constrained four-component mixture design. Unlike previous studies focused mainly on ternary binders or empirical proportioning, this work combined four-component formulation screening with age-dependent hydration characterization in one SS-BFS-OPC-DG framework. The 7 d compressive strength was selected as the modeled response because it provided a consistent early-age basis for comparing all 17 formulations within a practical testing period, whereas flexural strength was retained as a complementary experimental response. The compressive-strength mixture model was significant (*p* = 0.0044), with R^2^ = 0.692, adjusted R^2^ = 0.589, predicted R^2^ = 0.435, and adequate precision = 8.545. Its performance was evaluated using prediction indices, residual diagnostics, and an independent confirmation batch. The selected mass fractions of SS, BFS, OPC, and DG were 0.322, 0.429, 0.131, and 0.116, respectively. The confirmation batch achieved a 7 d compressive strength of 24.80 MPa, compared with the predicted value of 24.93 MPa. The model was therefore used only for formulation screening and interpolation within the investigated component ranges. The selected formulation reached compressive and flexural strengths of 32.6 and 7.6 MPa at 28 d, respectively. XRD, SEM, TG, and FTIR results collectively supported continued formation of hydration products and progressive matrix densification from 3 d to 28 d. The C-S-H gel index increased from 2.07% to 6.15%, while the number of large visible voids decreased and the continuity of the matrix improved. These results provide a constrained mixture-design route for screening high-solid-waste SS-BFS-OPC-DG binders.

## 1. Introduction

Steel slag is a major industrial solid waste produced by iron and steel enterprises and is characterized by relatively high alkalinity and good wear resistance [1]. Owing to its intrinsic properties and the steelmaking process, however, its recovery rate remains low and its synergistic utilization is limited [2]. In China, the accumulated stock of steel slag has exceeded 1 billion tonnes, with an annual output of approximately 100 million tonnes and a comprehensive utilization rate of about 30% [3]. Large-scale stockpiling occupies land and incurs continuing maintenance costs, while some hazardous constituents may enter soil and water bodies [4]. More effective routes for its coordinated utilization are therefore required.

Using steel slag in construction materials provides a route for consuming metallurgical solid waste and reducing the demand for virgin resources. Sun et al. evaluated pretreated steel slag for asphalt-pavement application and considered the leaching risk of hazardous substances [5]. Pan et al. compared steel slag and copper slag as raw materials for cementitious systems and discussed their performance differences and microstructural mechanisms [6]. Yang et al. investigated the workability, hydration, and carbonation of binders containing high volumes of lithium slag or steel slag [7]. Zhao et al. described a dissolution–precipitation hydration mechanism for steel slag activated through ion exchange with a layered activator [8]. Liu et al. examined an MgO-Na_2_CO_3_-activated recycled-fine-powder-slag system and reported changes in its mechanical and microstructural properties [9]. Wang et al. optimized an alkali-activated steel slag–slag binder and related its mechanical performance to microstructural development [10]. Ding et al. analyzed how steel slag content affected a one-step alkali-activated composite binder [11]. Sun et al. reported that triisopropanolamine and sulfite or sulfate acted synergistically in promoting the hydration of steel slag-based composite binders [12]. Fang and Song examined the influence of sodium-silicate content on the shrinkage and mechanical properties of MgO-activated slag [13]. Zhang et al. showed that CLDH seeding modified the hydration kinetics of a slag-based geopolymer through aluminosilicate phase engineering [14]. Jiang et al. tracked the early evolution of silicate species originating from waterglass in waterglass-activated slag pastes [15]. Zhang et al. characterized the hydration behavior and cementitious properties of steel slag from early to long curing ages [16]. Li et al. investigated mineral-phase evolution and hydration-activity enhancement of steel slag under NaOH activation [17]. Jin et al. examined interactions between slags and alkali–silicate activators and emphasized the role of Al-bearing phases [18]. Collectively, these studies show that steel slag reactivity can be altered by pretreatment, activator chemistry, sulfate regulation, and combination with other reactive solids. However, most of them address individual activation routes or binary and ternary binders rather than the coordinated proportioning of several industrial solid wastes.

More recent studies have moved toward multicomponent systems and quantitative formulation methods. Ye et al. improved the performance of steel slag mortar under low-temperature curing through alkali activation [19]. Sun et al. compared four activators in a desulfurization-ash-steel-slag-cement system and demonstrated that activator type affected early hydration [20]. Wang et al. applied XRD-based quantitative analysis to a quaternary solid-waste binder and its hydration products [21]. Ke and Zhang investigated the temperature sensitivity, hydration kinetics, and microstructural evolution of an SS-BFS-DG cementitious system [22]. Wang et al. evaluated concrete in which all-solid-waste materials replaced the binder, aggregates, or both [23]. Wang et al. studied a composite binder containing high volumes of steel slag, blast furnace slag, and desulfurization gypsum [24]. Kirchberger et al. found that sulfate, triethanolamine, and tartaric acid jointly affected the aluminate reaction in OPC [25]. Pinheiro et al. used response-surface methodology to optimize alkali-activated cement based on low-reactivity ladle-furnace slag [26]. Dvorkin et al. used a three-level four-factor experimental design and second-order polynomial regression models to develop a proportioning procedure for hybrid steel-basalt-fiber concrete [27]. Kim and Park discussed fractional simplex-centroid mixture designs for composition-dependent responses [28]. Myers et al. established a systematic framework for response-surface model construction, diagnosis, and optimization [29]. Mollah et al. demonstrated the use of FTIR spectroscopy to follow early Portland-cement hydration and associated silicate vibrations [30]. Li et al. reviewed the coordinated utilization of multiple solid wastes in ceramic foams, illustrating the broader material-design potential of multicomponent waste systems [31]. These studies provide a basis for component selection, statistical screening, and hydration characterization. Nevertheless, studies that combine mechanical-response screening, independent confirmation, and age-dependent hydration characterization within the same constrained SS-BFS-OPC-DG four-component design remain limited.

For multicomponent formulation, a constrained mixture design can reduce the required number of experimental formulations while covering the principal feasible blending region [28]. Response-surface principles further provide a structured basis for evaluating model significance, prediction performance, and residual behavior rather than relying on a single goodness-of-fit statistic [29]. Because component fractions are linked by a constant-sum constraint, the resulting model can describe blending effects and interaction trends directly. This approach is therefore suitable for screening high-solid-waste cementitious materials within prescribed component ranges.

In this study, SS, BFS, DG, and OPC were used to prepare a steel slag-based composite cementitious material. A constrained four-component mixture design was applied to relate component fractions to mechanical strength. Here, the term mixture model denotes the component–response relationship established within the bounded four-component mixture space. The 7 d compressive strength was selected as the modeled response and primary screening target, whereas flexural strength was retained as complementary experimental evidence. The selected formulation was independently confirmed, and its strength development was measured at 3 d, 7 d, 10 d, and 28 d. XRD, SEM, TG, and FTIR were subsequently combined to interpret hydration and microstructural evolution. Unlike previous studies focused mainly on binary or ternary steel slag-based binders, this study investigated an SS-BFS-OPC-DG quaternary system within a constrained four-component mixture space and combined formulation screening with age-dependent hydration characterization.

## 2. Materials and Methods

### 2.1. Materials

**Figure 1:**
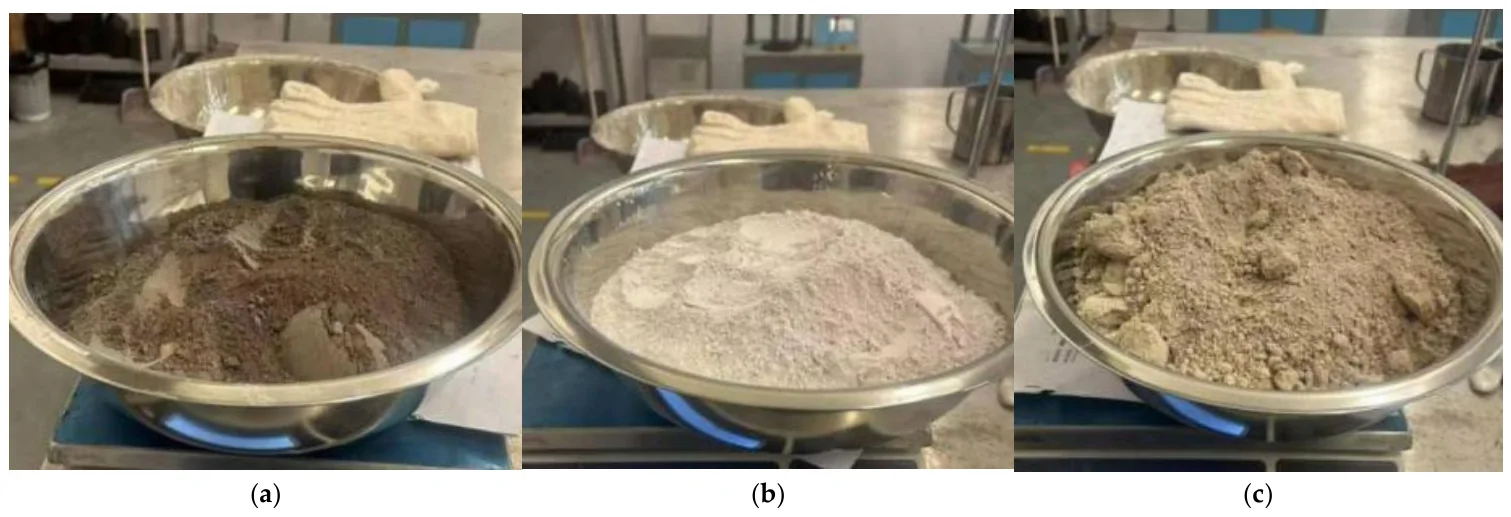
Appearances of the Raw Materials: (**a**) SS; (**b**) BFS; (**c**) DG.

The appearances of BFS, SS, and DG are shown in Figure 1. The technical properties of SS and BFS were measured in accordance with the Chinese standard JTG 3432-2024 [32] and are listed in Table 1. The measured values satisfy the technical requirements in the table. Specific surface area is used as an index of fineness, and the fluidity ratio is a relative flowability index measured according to JTG 3432-2024.

**Figure 2:**
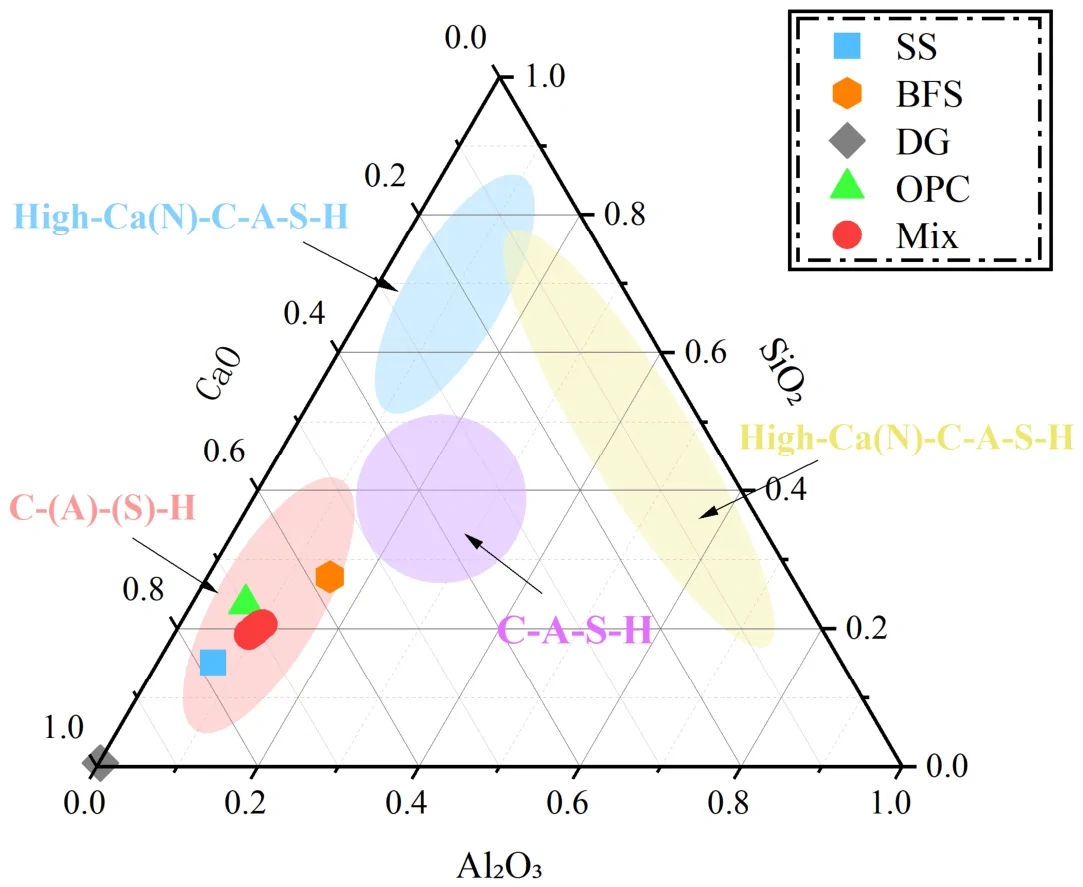
Superposition Diagram of Hydration Products of Solid Waste Materials.

The four components perform complementary chemical and physical roles. SS supplies calcium-bearing crystalline phases and alkaline constituents, BFS provides a comparatively reactive aluminosilicate glass phase, OPC supplies early hydration products and an alkaline pore solution, and DG provides sulfate for aluminate–sulfate reactions. Figure 2 places the principal raw-material oxides and anticipated hydration products in the CaO-Al_2_O_3_-SiO_2_ compositional framework to explain the component-selection rationale rather than to quantify any hydration product.

The component ranges were selected to retain steel slag as a major solid-waste constituent while accounting for the intended role of each material. SS and OPC supply calcium-bearing phases, BFS supplies reactive silicon- and aluminum-bearing species, and DG regulates sulfate availability. Under the adopted curing conditions, their combination may favor the formation of calcium silicate hydrates, calcium aluminosilicate hydrates, and AFt. The contribution of each component was evaluated in conjunction with the mixture model and the XRD, TG, FTIR, and SEM evidence.

### 2.2. Materials Characterization and Specimen Preparation

The chemical compositions of the raw materials, summarized in Table 2, were determined using a sequential wavelength-dispersive X-ray fluorescence spectrometer (ZSX Primus II, Rigaku Corporation, Tokyo, Japan). The samples were dried, ground, and directly pressed into pellets without adding a binder. XRF showed that SS contained 33.26% CaO and 13.27% total iron expressed as TFe. The CaO, SiO_2_, and Al_2_O_3_ contents of BFS were 29.77%, 14.25%, and 7.89%, respectively. DG was dominated by CaO and SO_3_, with respective proportions of 30.26% and 22.33%, consistent with its sulfate-bearing character. OPC contained 44.49% CaO. These compositions support the complementary roles assigned to the four components in the mixture design.

**Table 2:** Chemical composition of solid waste materials. (reproduced from [33]).

Composition	SS (%)	BFS (%)	DG (%)	OPC (%)
CaO	33.26	29.77	30.26	44.49
SiO_2_	6.42	14.25	0.16	9.16
Al_2_O_3_	2.94	7.89	0.07	3.33
TFe	13.27	0.21	0.04	2.50
SO_3_	0.25	1.00	22.33	1.64
Na_2_O	0.05	0.29	-	0.13
MgO	3.66	4.64	0.30	0.91
MnO	2.72	0.25	-	0.09

**Figure 3:**
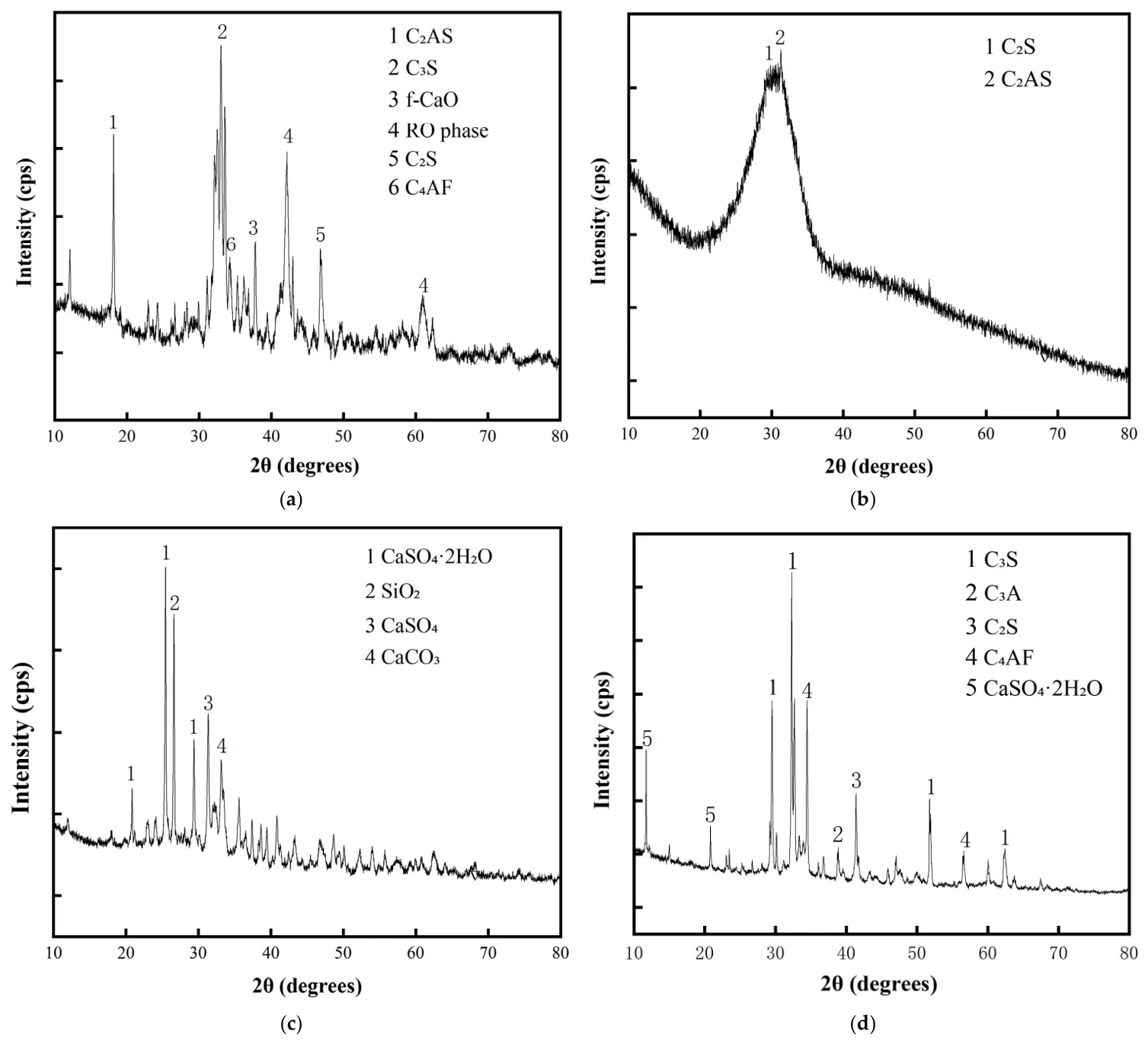
Phase Composition of the Raw Materials: (**a**) SS; (**b**) BFS; (**c**) DG; (**d**) OPC. Reproduced from [33].

The XRD patterns of SS, BFS, DG, and OPC are presented in Figure 3 and were used to identify the principal crystalline phases and broadened amorphous features. SS showed diffraction features associated with calcium silicate and iron-bearing phases. BFS displayed a broad diffuse background consistent with a comparatively high glass-phase content. DG was dominated by gypsum-related reflections, whereas OPC contained the common clinker phases.

**Figure 4:**
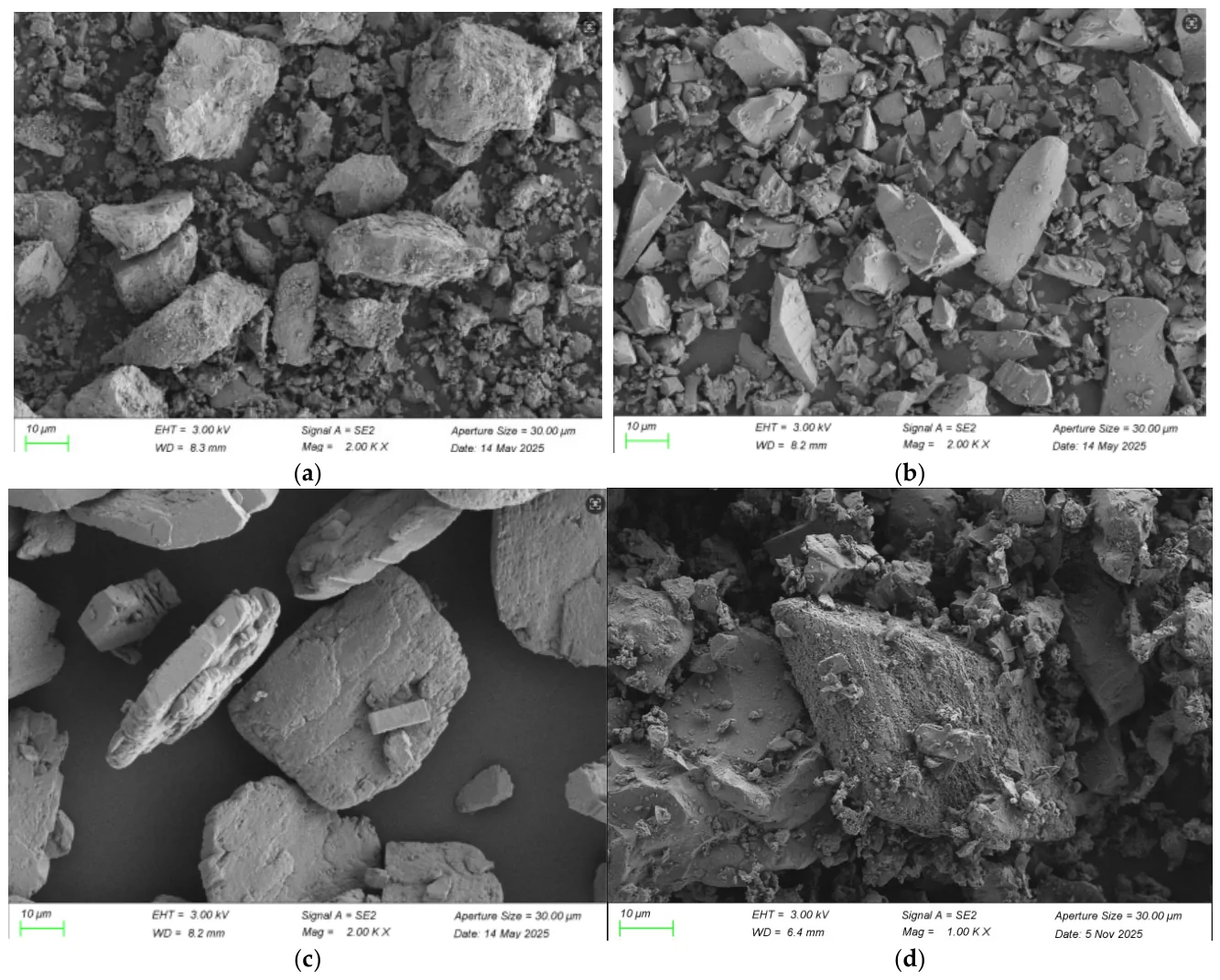
Representative SEM Morphologies of Raw-Material Particles: (**a**) SS, 2.00 kX; (**b**) BFS, 2.00 kX; (**c**) DG, 2.00 kX; (**d**) OPC, 1.00 kX.

Figure 4 presents representative particle morphologies of the four raw materials. SS particles are predominantly irregular and angular, with rough surfaces and visible pores or microcracks. BFS particles are finer and show a broader size distribution. DG occurs mainly as plate-like or columnar particles, whereas OPC contains irregular particles of different sizes and some agglomerates. Panels (a–c) were recorded at 2.00 kX and panel (d) at 1.00 kX; accordingly, the images are used to describe typical morphology rather than for direct quantitative comparison of particle size or pore dimensions.

Each panel represents one individual raw material rather than a phase distribution in a reacted matrix. Although the nominal magnification of the OPC image differs from those of the other panels, all panels contain scale bars of the same length. The images are therefore used only for the qualitative characterization of typical particle morphology rather than for direct quantitative comparison of particle dimensions or pore characteristics, while the associated mineralogical and chemical interpretations are based on the XRD and XRF results.

### 2.3. Experimental Method

#### 2.3.1. Constrained Four-Component Mixture Design

A constrained four-component mixture design was used because the mass fractions of SS, BFS, OPC, and DG sum to 100% [28]. Damasceno et al. demonstrated that mixture-design models can represent composition-dependent responses within constrained multicomponent regions, although their material system differed from the present cementitious binder [34]. Response-surface methodology has also been used to optimize composition-dependent particulate processes [35]. The component ranges in the present study were selected with reference to common contents reported for steel slag-based composite binders, the intended function of each component, and the high-solid-waste objective of this study. SS, the principal material of interest, was set at 30–40%; BFS, the main reactive solid-waste constituent, at 40–50%; OPC at 10–15% to provide early hydration products and an alkaline reaction environment; and DG at 10–15% to supply sulfate and regulate aluminate–sulfate reactions. These independent bounds define a practically preparable sub-region of the four-component mixture space in which a regional candidate composition was sought. The constrained mixture design, model fitting, statistical diagnostics, and numerical optimization were performed using Design-Expert 13 (Stat-Ease, Inc., Minneapolis, MN, USA). Equation (1) gives the polynomial hierarchy considered for this mixture:

(1) $$Y=\sum \beta_{i}x_{i}+\sum \beta_{\mathrm{ij}}x_{i}x_{j}+\sum \beta_{\mathrm{ijk}}x_{i}x_{j}x_{k}{+\beta }_{1234}x_{1}x_{2}x_{3}x_{4}$$

In Equation (1), Y is the response and the beta terms are the coefficients of the component and interaction terms [29]. The primary scientific focus was the SS-DG blending effect because SS supplies calcium-, silicon-, and aluminum-bearing phases, whereas DG regulates sulfate availability; their balance may therefore influence aluminate–sulfate reactions and mechanical performance. The model first retained A, B, C, and D to preserve the complete four-component mixture basis, where A, B, C, and D represent SS, BFS, OPC, and DG, respectively. AD and the other candidate interaction terms were then screened using overall significance, adjusted and predicted R^2^, PRESS, adequate precision, residual behavior, and the residual degrees of freedom.

Seventeen formulations were selected within these component constraints to cover the principal boundary and interior regions of the feasible mixture space (Table 3). All formulations contained the four components so that their combined mechanical response could be investigated. The 7 d compressive strength was selected as the modeled response because it provided a consistent early-age comparison for all 17 formulations within a practical testing period and reflected the load-bearing response used for formulation screening. For numerical optimization, the four component fractions were constrained to sum to one, and candidate solutions were generated only within the investigated mixture region. The highest-ranked feasible composition was subsequently evaluated in an independent confirmation batch. Tests at 3 d, 10 d, and 28 d were used to examine the strength development of the selected formulation and were not included in fitting the 7 d model.

#### 2.3.2. Testing Methods

Water, SS, BFS, OPC, and DG were weighed according to the designed formulations and mixed before standard sand was added. The mortar was cast into 40 mm × 40 mm × 160 mm prisms, cured for 24 h at 20 ± 2 °C and for a relative humidity of at least 95%, demolded, and then water-cured to the specified ages. Three prisms were tested in flexure for each formulation. The two halves produced after fracture of each prism were used for compression, giving six compressive specimens per formulation. Samples for microstructural characterization were taken from the interior of the hardened material and dried at 40 °C for 24 h.

The 7 d flexural strength was measured using a DKZ-5000 electric flexural testing machine (Wuxi Jianyi Instrument Machinery Co., Ltd., Wuxi, China), followed by compressive testing using a YAW-300 microcomputer-controlled electro-hydraulic servo compression testing machine (Shenzhen Suns Technology Stock Co., Ltd., Shenzhen, China). Flexural and compressive results are the means of three and six specimens, respectively. XRD samples were ground to pass a 200-mesh sieve and scanned using an X-ray diffractometer (D8 ADVANCE, Bruker AXS GmbH, Karlsruhe, Germany) from 10° to 80° (2θ) at 5°/min. SEM specimens, approximately 5 mm × 5 mm × 1 mm, were dried and platinum-coated using a JFC-1600 automatic fine coater (JEOL Ltd., Tokyo, Japan). The hardened matrices were observed using a field-emission scanning electron microscope (Sigma 300, Carl Zeiss Microscopy GmbH, Jena, Germany) at an accelerating voltage of 3.0 kV and a working distance of approximately 9.4 mm; secondary-electron images were acquired using an SE2 detector at the same nominal magnification. For TG analysis, 10–15 mg of powder was placed in an alumina crucible and analyzed using an STA 449 F3 Jupiter simultaneous thermal analyzer (NETZSCH-Gerätebau GmbH, Selb, Germany). The samples were heated to 800 °C at 10 °C/min under nitrogen flowing at 50 mL/min. FTIR specimens were dried, ground, mixed with KBr, pressed into pellets, and analyzed using a TENSOR II Fourier transform infrared spectrometer (Bruker Optics GmbH & Co. KG, Ettlingen, Germany). The spectra were collected from 400 to 4000 cm^−1^ at a resolution of 4 cm^−1^ with 32 scans.

### 2.4. Semi-Quantitative Analysis of Thermogravimetric Data

A characteristic-temperature-interval mass-loss method was used for semi-quantitative comparison of the TG data. All curves were normalized to 100% at 50 °C. Mass loss from 50 to 380 °C was used to calculate a dry-basis C-S-H gel index. Mass losses at 450–550 °C and 600–750 °C were used to estimate the relative contents associated with Ca(OH)_2_ dehydroxylation and CaCO_3_ decomposition, respectively. Because the processes overlap, the derived values are used only for semi-quantitative comparison among samples tested under identical conditions.

The C-S-H gel index was estimated from the mass loss between 50 and 380 °C and corrected to the dry basis at 800 °C using Equation (2). The calculated value is used as a comparative semi-quantitative index rather than as an absolute phase content:

(2) $$\mathrm{CSH}=\frac{m_{50}-m_{380}}{m_{800}}\times 100\%$$

where *m*_50_, *m*_380_, and *m*_800_ are the normalized masses at 50 °C, 380 °C, and 800 °C, respectively.

Calcium hydroxide (CH) content was calculated from the mass loss caused by Ca(OH)_2_ dehydroxylation between 450 and 550 °C. The reaction is Ca(OH)_2_(s) → CaO(s) + H_2_O(g), and the theoretical H_2_O mass fraction is 18/74, or approximately 0.243. The CH content is given by Equation (3):

(3) $$\mathrm{CH}=\frac{\Delta m_{450–550}}{0.243}\times 100\%$$

where Δ*m*_450–550_ is the normalized mass loss (on an 800 °C dry basis) in the 450–550 °C interval.

Calcium carbonate (CaCO_3_) content was derived from the mass loss caused by CaCO_3_ decomposition between 600 and 750 °C. The reaction is CaCO_3_(s) → CaO(s) + CO_2_(g), and the theoretical CO_2_ mass fraction is 44/100, or 0.44. The CaCO_3_ content is calculated using Equation (4):

(4) $${\mathrm{CaCO}}_{3}=\frac{\Delta m_{600–750}}{0.44}\times 100\%$$

where Δ*m*_600–750_ is the normalized mass loss (on an 800 °C dry basis) in the 600–750 °C interval.

Because the dehydration signals of AFt and C-(A)-S-H overlap, precise deconvolution is not possible from the TG curves alone. The calculated values are therefore semi-quantitative and are used only to compare samples tested under the same conditions.

## 3. Results and Discussion

### 3.1. Analysis of Mechanical Response Model

**Figure 5:**
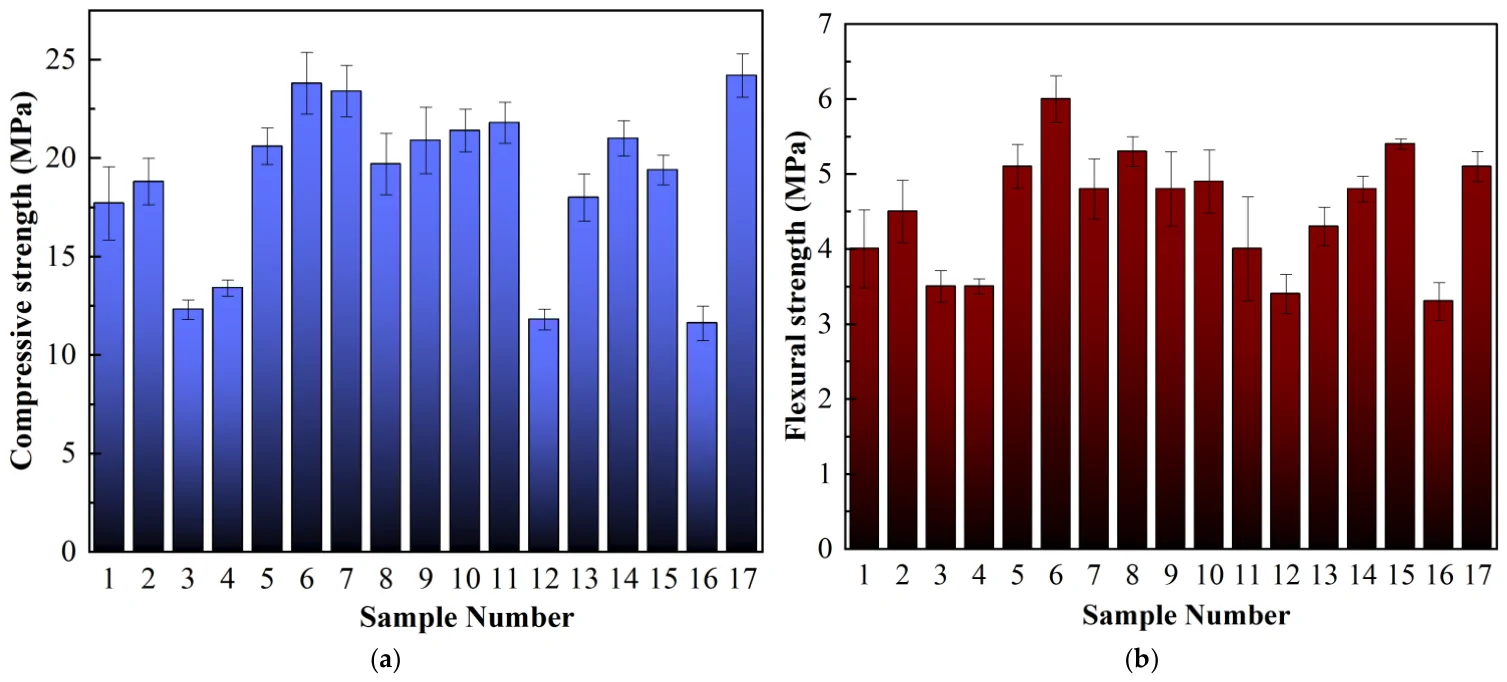
Mechanical Strength of Cementitious Materials at 7 d: (**a**) Compressive Strength (n = 6); (**b**) Flexural Strength (n = 3). Values are mean ± SD, and error bars represent one SD.

Figure 5 presents the 7 d compressive and flexural strengths of the 17 formulations, with A1–A17 corresponding to Table 3. Each bar is a mean and each error bar represents the standard deviation. The response values vary appreciably across the constrained composition space, confirming that mechanical performance depends on redistribution among the four constituents. A17 had the highest measured mean compressive strength (24.17 MPa), whereas A6 had the highest measured mean flexural strength (6.00 MPa). Compressive strength was selected as the primary modeling response because it directly represents the load-bearing capacity used for formulation screening and was based on six half-prism measurements per formulation. Its mean coefficient of variation was 5.40%. By comparison, flexural strength was obtained from three prisms and showed greater overall dispersion, with a mean coefficient of variation of 10.70%; particularly high variability occurred for A7 and A11. The choice of compressive strength therefore reflects both engineering relevance and more stable repeatability across the complete design, rather than the behavior of any single formulation. Flexural strength is retained as complementary evidence. Table 4 reports the mean, standard deviation, and coefficient of variation for both responses.

Table 4 summarizes the mean, standard deviation, and coefficient of variation of the 7 d flexural and compressive results. The statistics are based on three flexural specimens and six compressive specimens for each formulation.

A four-component mixture model was fitted to the 7 d compressive-strength data in Design-Expert following established response-surface principles [29]. A related response-surface application also demonstrates the use of statistical formulation screening in composition-dependent particulate systems [35]. Composition-sensitive mechanical responses have been reported for waste-based cementitious composites containing multiple modifiers [36]. Ding et al. further showed that activator formulation, represented by water-glass modulus, can materially affect the mechanical properties of geopolymer recycled-aggregate concrete [37]. Candidate terms in the present model were screened using model significance, were adjusted and predicted R^2^, adequate precision, PRESS, residual behavior, and residual degrees of freedom. The retained terms A, B, C, and D maintain the complete SS-BFS-OPC-DG mixture basis, while AD describes the targeted SS-DG blending effect.

**Figure 6:**
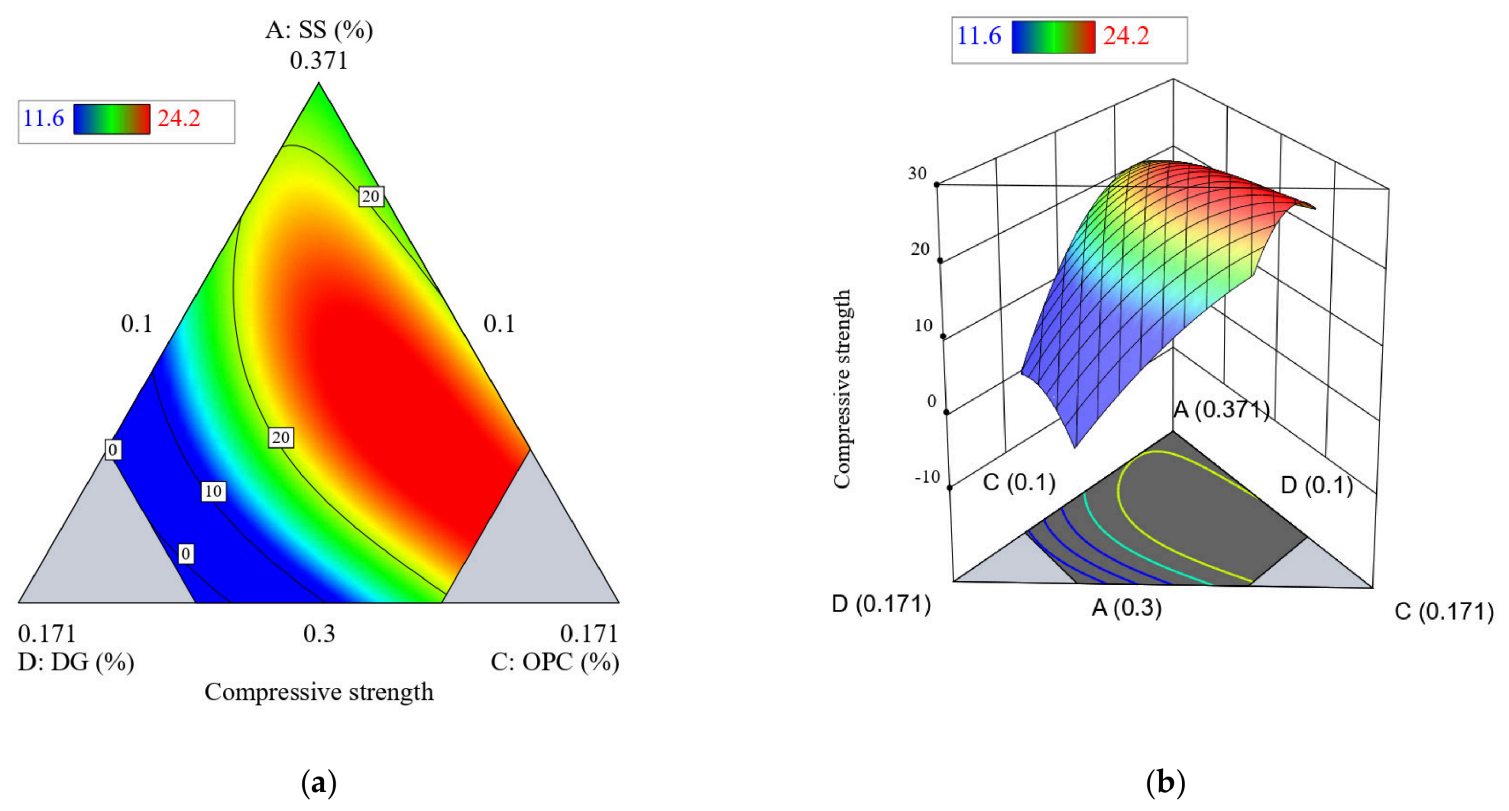
Predicted 7 d Compressive-Strength Response in the Four-Component Mixture Space at BFS = 0.429: (**a**) Contour Plot; (**b**) Three-Dimensional Response Surface.

Figure 6 shows that, with the BFS mass fraction fixed at 0.429, the predicted compressive strength does not vary linearly with SS, OPC, or DG. Instead, a high-response region occurs within the feasible mixture space. This indicates that compressive strength depends on the balance among the component proportions rather than on an increase in any single component. SS provides calcium-bearing reactive phases, whereas DG regulates sulfate availability. When their proportions deviate from an appropriate balance, the reaction compatibility between calcium-bearing phases and sulfate may be weakened, resulting in lower predicted strength. The curvature of the response surface is consistent with the significant SS–DG interaction term, indicating a non-additive blending effect on compressive strength. The selected composition lies within this high-response region, suggesting that its advantage arises from the coordinated balance of the four components under the imposed constraints.

**Figure 7:**
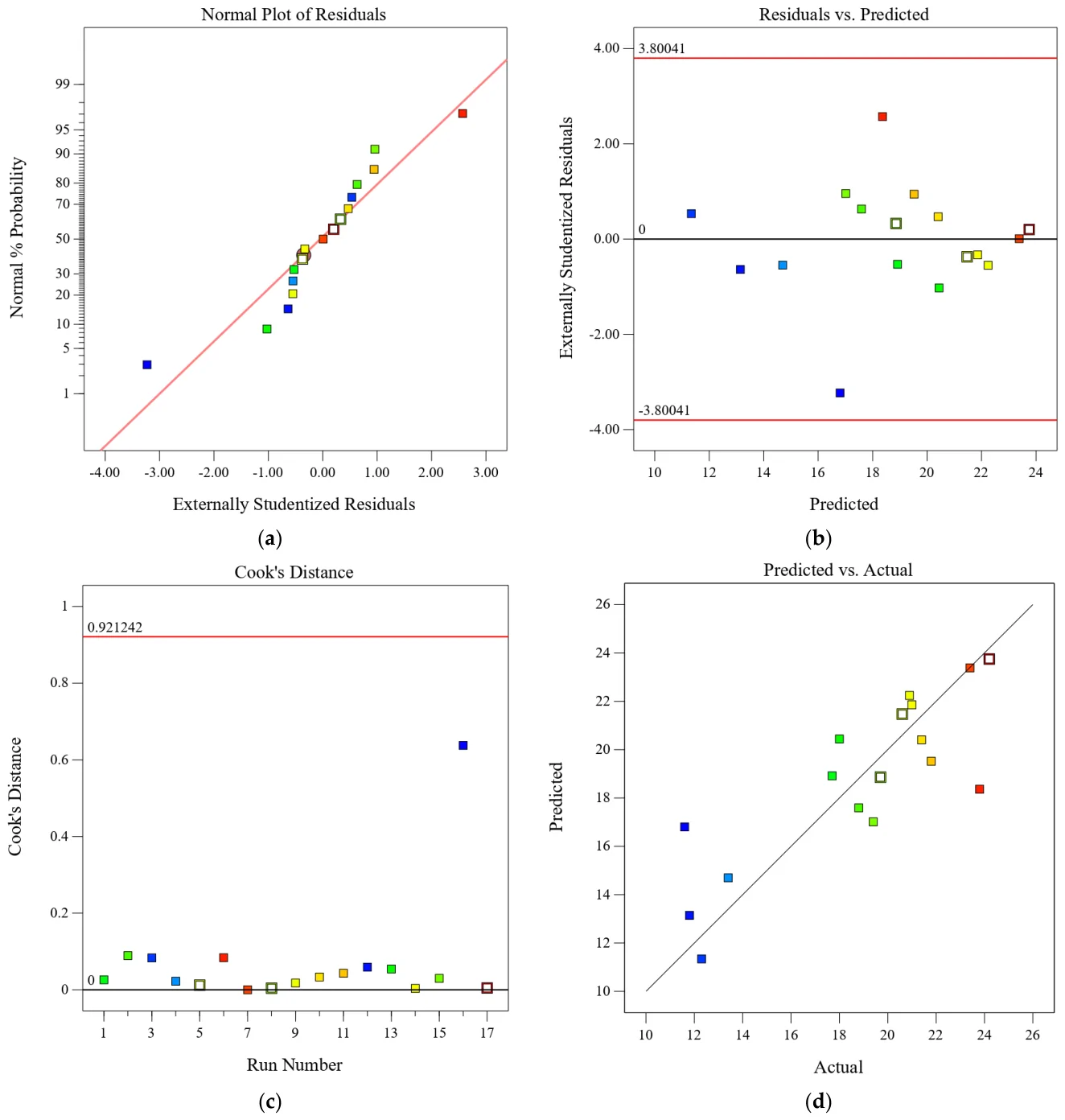
Diagnostic Results of the Four-Component Mixture Model for 7 d Compressive Strength: (**a**) Normal Probability Plot of Externally Studentized Residuals; (**b**) Externally Studentized Residuals versus Predicted Values; (**c**) Cook’s Distance; (**d**) Predicted versus Actual Values.

The final model contains A, B, C, D, and AD. The overall model was significant (*p* = 0.0044), and the SS-DG interaction term AD was also significant (*p* = 0.0147). The model yielded R^2^ = 0.692, adjusted R^2^ = 0.589, predicted R^2^ = 0.435, adequate precision = 8.545, and PRESS = 157.59. The corresponding diagnostic plots and statistical indicators are presented in Figure 7 and Table 5, respectively.

**Table 5:** Statistical Indicators of the Four-Component Mixture Model for Compressive Strength.

Response	Model *p*-Value	R^2^	Adjusted R^2^	Predicted R^2^	Adequate Precision	AD-Term *p*-Value
	0.0044	0.692	0.589	0.435	8.545	0.0147

No universal R^2^ threshold alone establishes the adequacy of a response-surface or mixture model; model significance, prediction statistics, residual behavior, and intended use should be considered together [29]. In the present model, the significant model *p*-value and adequate precision indicate that the dominant composition-related signal can be distinguished from experimental noise. The difference between adjusted R^2^ and predicted R^2^ is 0.154, showing no severe mismatch between fitted and excluded-observation performance, although the moderate predicted R^2^ also indicates that part of the local variation remains unresolved. This interpretation is consistent with the diagnostics: the normal-probability plot shows no serious departure from linearity, the residuals exhibit no systematic curvature over the predicted range, and every Cook’s distance lies below the reference limit. The unexplained variation is therefore more consistent with experimental dispersion and local effects not represented by the retained terms than with systematic model bias or a single controlling observation. Accordingly, the model is used to compare feasible formulations, interpret the SS-DG blending effect, and screen candidate compositions within the investigated region; extrapolation beyond the prescribed component ranges or to different raw-material sources requires additional validation.

### 3.2. Optimization and Verification of Design Scheme

**Figure 8:**
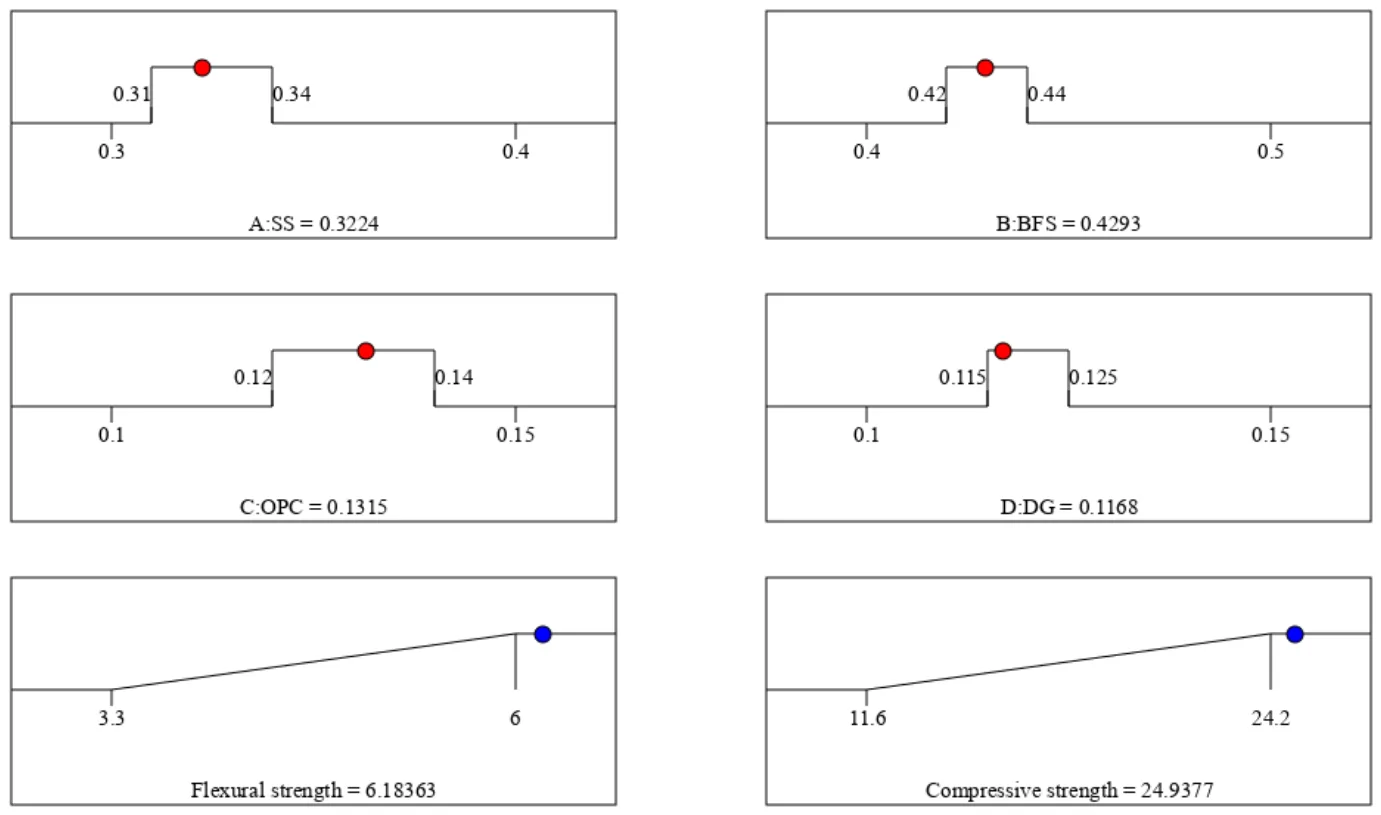
Design-Expert Output for the Selected Regional-Optimum Composition and Predicted Mechanical Responses. Red points indicate the selected component fractions, whereas blue points indicate the corresponding predicted response values.

Under the component constraints defined in Section 2.3.1, Design-Expert searched the investigated four-component mixture region with 7 d compressive strength as the primary response. The selected composition is interpreted as a regional candidate screened within the prescribed component ranges, and its suitability was evaluated using an independent confirmation batch. Figure 8 retains the two mechanical outputs generated by the software: compressive strength was used to select the candidate formulation, whereas flexural strength is shown only as a complementary performance output and is not used to establish the statistical reliability of the compressive-strength model.

**Figure 9:**
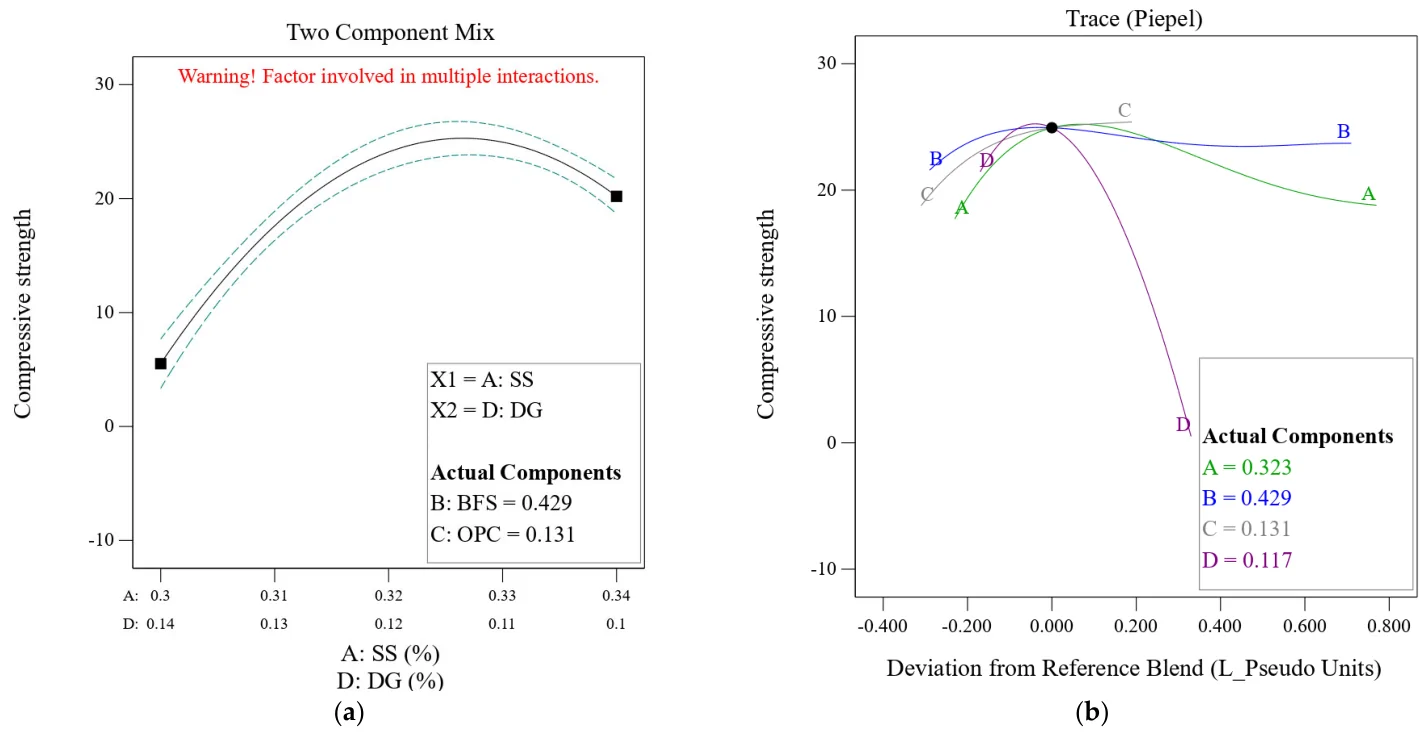
Predicted Component Effects around the Selected Composition: (**a**) SS-DG Two-Component Mixture Plot at BFS = 0.429 and OPC = 0.131; (**b**) Piepel Trace Plot at the Reference Blend. In panel (**a**), the solid black curve represents the model-predicted compressive strength, the cyan dashed curves represent the 95% confidence limits of the mean prediction, and the black squares denote experimental design points. In panel (**b**), the black dot denotes the reference blend, and the colored curves show the predicted response to deviations of each component from this blend.

The selected SS, BFS, OPC, and DG mass fractions were 0.322, 0.429, 0.131, and 0.116, respectively, with a predicted 7 d compressive strength of 24.93 MPa. This solution represents a balance among the four components under the constant-sum constraint rather than the maximization of an individual constituent. In Figure 9a, BFS and OPC are fixed at 0.429 and 0.131, and the predicted response follows a concave SS-DG blending curve. Strength increases as the composition approaches an intermediate SS-DG balance near the selected formulation and decreases toward either end of the feasible path. The response therefore cannot be interpreted as an independent monotonic effect of SS or DG; it is consistent with the statistically significant AD term and indicates a non-additive blending relationship between these components. In the Piepel trace plot (Figure 9b), all component traces converge at the reference blend. Departures from this point generally do not improve the predicted response, with the DG direction showing the steepest loss and the SS direction also declining when SS is increased beyond the reference balance; the BFS and OPC directions change more moderately over their feasible paths. Together, the two panels locate the selected formulation within a local high-response region of the constrained four-component space and clarify that its performance arises from coordinated component balance.

**Figure 10:**
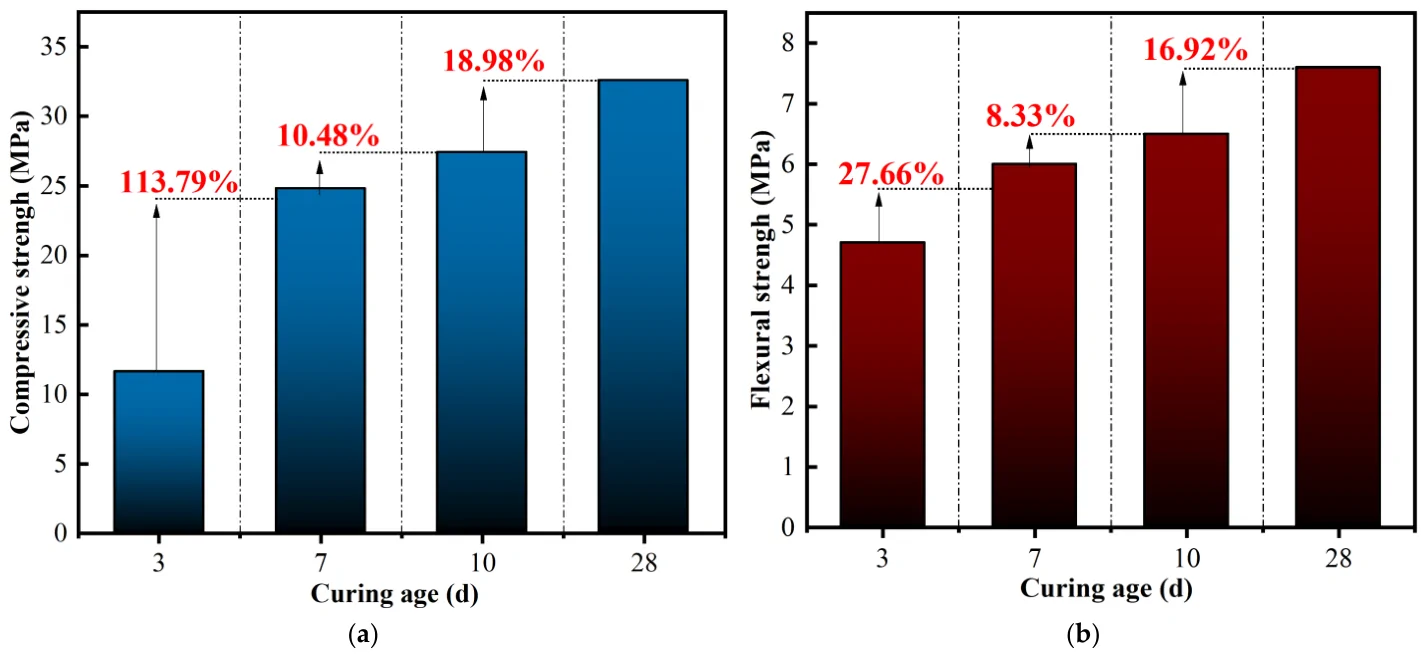
Mechanical Strength at Different Curing Ages: (**a**) Compressive Strength; (**b**) Flexural Strength.

An independent batch with the selected composition was prepared and excluded from model fitting. Its measured 7 d compressive strength was 24.80 MPa, compared with the predicted value of 24.93 MPa, giving a relative error of approximately 0.5%. This close local agreement supports use of the model for screening a candidate in the investigated region, but one confirmation point cannot establish generally high predictive accuracy. Reliability must therefore be interpreted together with the analysis of variance, prediction indices, and residual diagnostics in Section 3.1. The measured 7 d flexural strength was 6.02 MPa and is reported as a complementary property rather than as a modeled response. Additional tests at 3 d, 10 d, and 28 d showed continued strength development, as shown in Figure 10; the 28 d compressive and flexural strengths reached 32.6 and 7.6 MPa, respectively, providing the mechanical basis for subsequent microstructural interpretation without being included in fitting the 7 d mixture model.

### 3.3. Preliminary Raw-Material Cost Assessment

To preliminarily evaluate the economic feasibility of the selected composition, the raw-material cost was estimated using 1 t of dry binder as the functional unit. Representative unit prices of SS, BFS, OPC, and DG were assumed to be CNY 100/t, CNY 170/t, CNY 275/t, and CNY 40/t, respectively, and a binder containing 100% OPC was used as the cost reference. Based on the normalized quantities of the four components, their respective cost contributions were CNY 32.26/t, CNY 73.08/t, CNY 36.11/t, and CNY 4.65/t. The total raw-material cost of the selected binder was approximately CNY 146.1/t, about 47% lower than the CNY 275.0/t OPC reference (Table 6). Because only raw-material costs were considered, this calculation represents a preliminary cost comparison rather than a complete economic assessment.

### 3.4. Hydration Products and Microstructural Evolution

XRD, SEM, TG, and FTIR were combined to analyze the hydration products and microstructural development of the selected steel slag-based cementitious material. The methods were interpreted as complementary evidence because no single technique can independently quantify all crystalline, poorly crystalline, and morphological features.

#### 3.4.1. XRD Analysis

**Figure 11:**
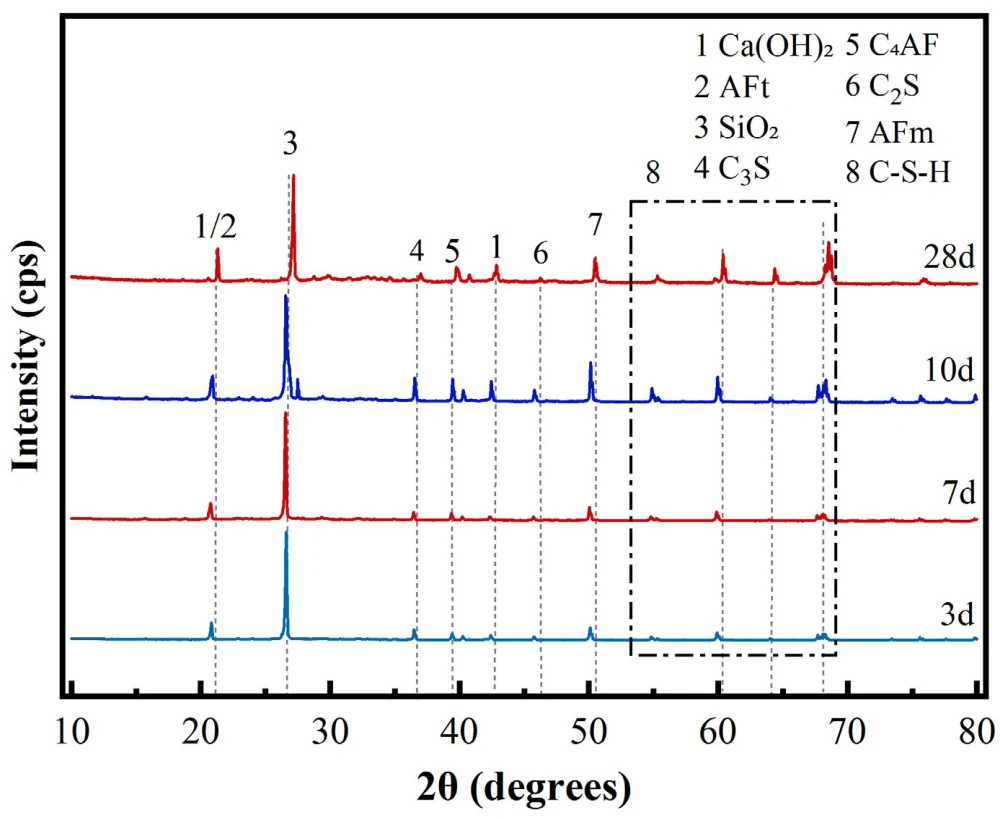
XRD Patterns of Cementitious Materials at Different Ages. The dashed rectangle highlights the broad C-S-H-related diffraction region used for qualitative comparison.

The XRD patterns at different ages are shown in Figure 11. Previous studies demonstrate that the hydration of slag-rich binders is sensitive to chemical regulation. Zhai et al. reported that triethanolamine dosage altered the hydration of blast-furnace-slag cement [38]. Xin et al. linked the action of CO_2_-absorbed triethanolamine solutions to the combined effects of cement hydration and carbonation [39]. Gu et al. examined the synergistic activation of desulfurization gypsum by calcium carbide residue and fly ash [40]. Jing et al. related steel slag utilization in mortar to changes in hydration and microstructure [41]. In the present system, reflections associated with AFt and CH are detectable at 3 d, which is consistent with the occurrence of clinker hydration and sulfate–aluminate reactions. Residual crystalline phases from steel slag and clinker remain prominent, while the broadened diffuse background in the silicate region is consistent with poorly crystalline or amorphous silicate and aluminosilicate products. Together, these features indicate the qualitative coexistence of crystalline hydrates, residual raw-material phases, and poorly crystalline reaction products at 3 d.

From 3 d to 7 d, changes in the AFt- and CH-related reflections and in the broadened background are consistent with continued sulfate–aluminate and calcium-bearing reactions. Residual clinker and steel slag reflections remain identifiable, showing that unreacted crystalline constituents persist, whereas the broadened background supports the continued presence and formation of poorly crystalline hydration products.

At 10 d, the apparent intensities of several residual reflections decrease while the broadened amorphous contribution persists. Considered together with the increase in the TG-derived C-S-H gel index from 2.92% at 7 d to 3.72% at 10 d, these observations are consistent with continuing hydration and the development of poorly crystalline products. Chen et al. showed that carbonation treatment changes the properties of steel slag used in cementitious materials [42]. Tian et al. reported coupled hydration–carbonation behavior when steel slag was activated by triethanolamine and CO_2_ [43]. These findings support considering carbonation-related contributions when interpreting later-age phase changes. At 28 d, AFt-related reflections, residual crystalline phases, and the broadened background still coexist, indicating continued hydration and the gradual development of a mature multiphase matrix.

#### 3.4.2. SEM Analysis

**Figure 12:**
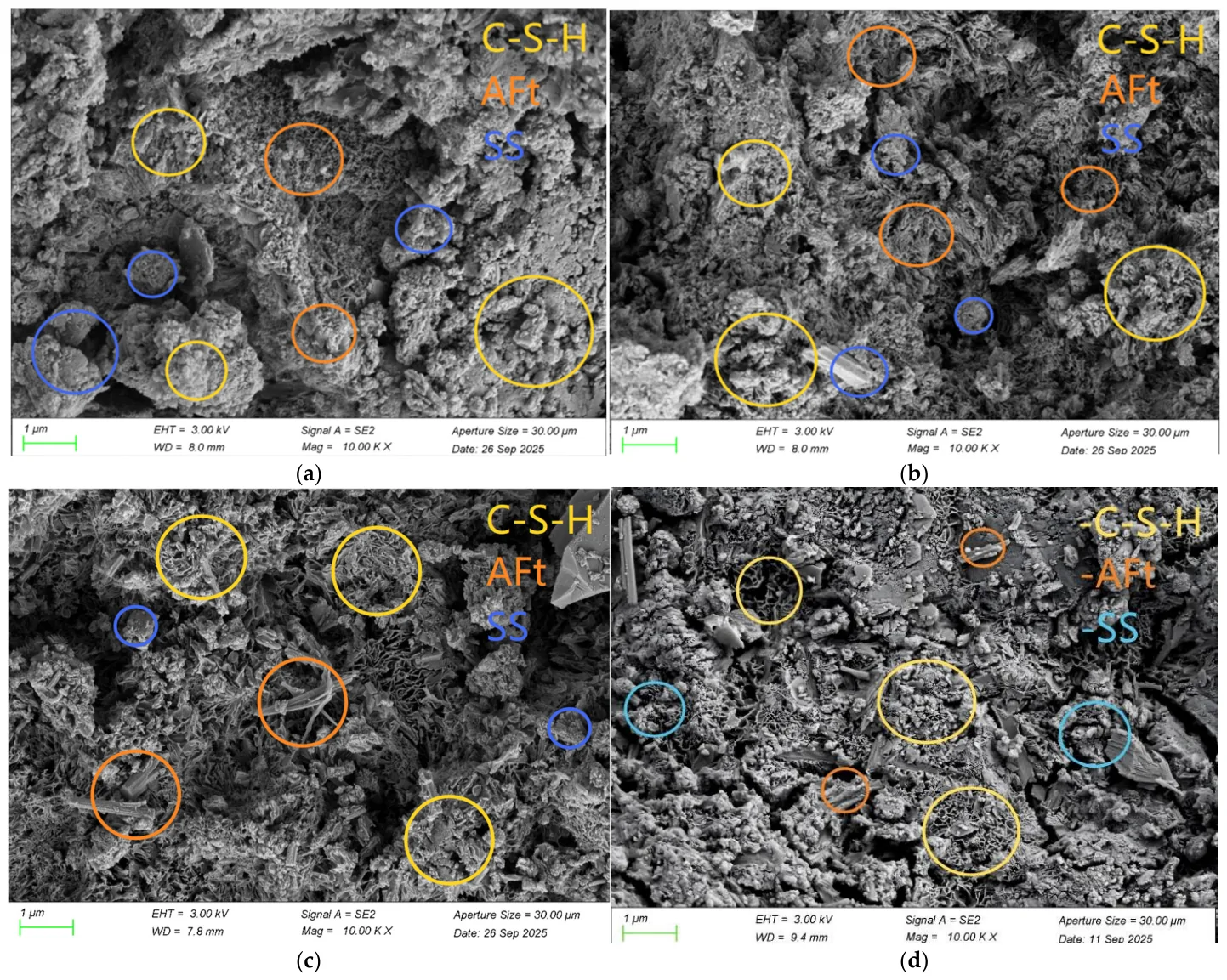
SEM Micrographs of the Hardened Matrix at the Same Magnification: (**a**) 3 d; (**b**) 7 d; (**c**) 10 d; (**d**) 28 d.

Figure 12 compares the hardened matrices at the same nominal magnification. Microstructural development has also been used to interpret the performance of zero-clinker binders prepared from industrial wastes [44]. Li et al. similarly related the performance of alkali-activated binders containing coal-gangue-derived slag to their reaction products and matrix development [45]. In the present material, the matrix at 3 d is discontinuous, with numerous angular residual particles, open voids, and locally distributed needle-like and flocculent gel-like products. The limited continuity of the reaction products indicates that an interconnected particle-bridging network has not yet developed. The exposed particles and open voids can act as mechanically weak regions, which is consistent with the lower early-age strength.

At 7 d, more reaction products cover particle surfaces, fill interparticle spaces, and bridge neighboring particles. The reduction in large visible voids and the improved matrix continuity indicate that filling and bridging increasingly contribute to load transfer.

At 10 d, flocculent gel-like and plate-like regions form a more connected matrix, and residual particles are increasingly embedded in surrounding products. This development reduces structural discontinuities and produces more continuous load-transfer paths. At 28 d, the matrix is denser and more continuous, with fewer large voids and exposed residual particles. The persistence of some unreacted particles shows that the later strength gain does not require the complete reaction of all raw materials; instead, it is associated mainly with greater matrix continuity and improved interparticle connection.

The C-(A)-S-H, AFt, and SS labels in Figure 12 combine morphological observations with the XRD, TG, and FTIR evidence and are used as tentative interpretations of the microstructural features.

#### 3.4.3. TG Analysis

**Figure 13:**
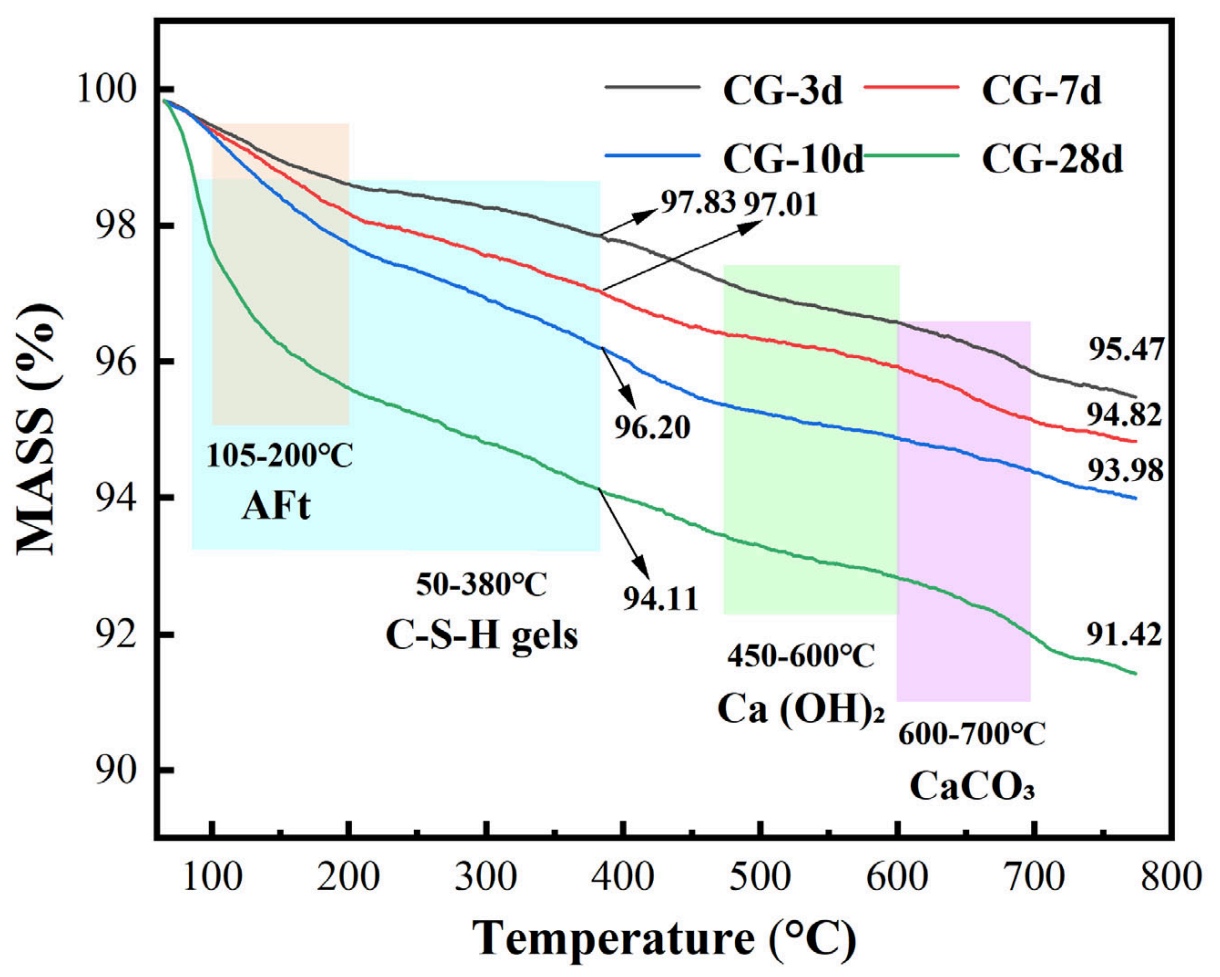
TG Curves of Cementitious Materials at Different Curing Ages.

The TG curves at different curing ages are shown in Figure 13. The 50–380 °C mass-loss interval was used to calculate the comparative C-S-H gel index. The 450–550 °C interval is associated mainly with Ca(OH)_2_ dehydroxylation, whereas 600–750 °C is associated mainly with CaCO_3_ decarbonation. Because the processes overlap, the calculated values are interpreted semi-quantitatively across ages rather than as absolute contents of individual phases.

The semi-quantitative indices calculated using the procedure in Section 2.4 are summarized in Table 7.

The C-S-H gel index increased sequentially from 2.07% at 3 d to 2.92% at 7 d, 3.72% at 10 d, and 6.15% at 28 d. Studies of gypsum-based composite cementitious materials also show that hydration-related mass-loss regions must be interpreted together with phase and microstructural evidence [46]. In the present system, the increase in the C-S-H gel index, particularly from 10 d to 28 d, is consistent with the continued development of hydration products during later curing and agrees with the improvement in matrix continuity observed by SEM. The calculated CH index decreased from 2.54% at 3 d to 1.48% at 7 d and then increased to 2.03% at 10 d and 2.55% at 28 d. Because these values are derived from fixed temperature intervals and may also be affected by overlapping mass-loss processes, sampling variation, and baseline selection, the non-monotonic CH trend is not assigned to a single reaction mechanism. It may reflect the combined effects of CH formation and consumption, but the present data do not separate those contributions quantitatively. The CaCO_3_ index decreased from 2.32% at 3 d to 0.57% at 7 d and then increased to 1.70% and 1.80% at 10 d and 28 d, respectively. This index can include contributions from both initial carbonates and curing-related carbonation and therefore is not interpreted as a direct measure of carbonation alone. The clearest age-dependent TG result is the increase in the comparative C-S-H gel index.

#### 3.4.4. FTIR Analysis

**Figure 14:**
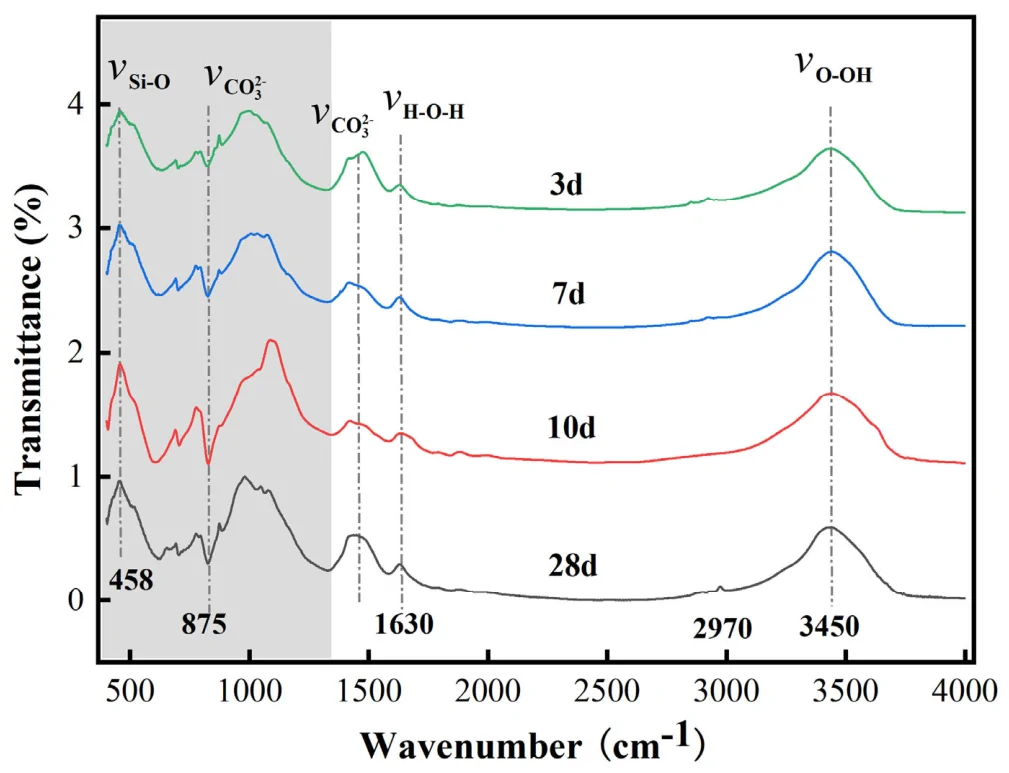
FTIR Spectra of Cementitious Materials at Different Curing Ages. The gray-shaded area highlights the fingerprint region containing the principal Si–O and carbonate-related vibration bands.

Figure 14 shows the FTIR spectra from 400 to 4000 cm^−1^. The broad band near 3450 cm^−1^ is assigned to hydrogen-bonded O-H stretching in adsorbed water and hydroxyl-bearing phases, and the band near 1630 cm^−1^ is assigned to H-O-H bending. The band near 458 cm^−1^ is associated with Si-O or O-Si-O bending, consistent with silicate bending bands reported for hydrated cementitious systems [30]. Bands near 875 cm^−1^ and in the 1400–1500 cm^−1^ region are associated with carbonate vibrations. The broad 900–1100 cm^−1^ region contains Si-O-T vibrations and sulfate-related contributions, where T denotes Si or Al. Salha et al. showed that Al can participate in oligomerization within cement-forming silicate systems [47]. Subsequent work by Salha et al. further related Al-driven structural organization and disorder to cement functionality [48]. These studies support treating the Si-O-T region as a combined silicate–aluminate bonding environment rather than assigning it to a single crystalline phase. No distinct absorption band is resolved near 2970 cm^−1^; consequently, no C-H assignment is made for this inorganic cementitious system. Changes in the O-H and Si-O-T regions with curing age are consistent with continued evolution of water-bearing hydrates and silicate/aluminosilicate bonding environments. The carbonate bands agree qualitatively with the TG-derived CaCO_3_ index, but they cannot by themselves distinguish initial carbonate from curing-related carbonation.

Taken together, the four characterization methods provide complementary evidence. XRD shows the persistent coexistence of residual crystalline phases and a broadened contribution from poorly crystalline products, TG shows an increase in the comparative C-S-H gel index, FTIR records changes in hydration-related groups and the Si-O-T bonding environment, and SEM reveals progressive void filling and particle connection. These observations are collectively consistent with continued hydration and improved matrix continuity from 3 d to 28 d. Because each method has intrinsic limitations, the evidence does not quantify individual phase contents or separate the absolute contributions of SS, BFS, OPC, and DG.

## 4. Conclusions

A constrained four-component mixture design was used to investigate an SS-BFS-OPC-DG cementitious material. A mixture model was established for 7 d compressive strength, while flexural strength was retained as a complementary experimental response.

Within these ranges, the selected SS, BFS, OPC, and DG mass fractions were 0.322, 0.429, 0.131, and 0.116, respectively. The independent confirmation batch achieved a 7 d compressive strength of 24.80 MPa. The selected material reached compressive and flexural strengths of 32.6 and 7.6 MPa at 28 d, respectively.

Using the assumed representative unit prices, the preliminary raw-material cost of the selected binder was estimated at CNY 146.1/t, approximately 47% below that of the 100% OPC reference.

The four-component compressive-strength model was significant, with *p* = 0.0044, R^2^ = 0.692, adjusted R^2^ = 0.589, predicted R^2^ = 0.435, and adequate precision = 8.545. The SS-DG interaction term was significant (*p* = 0.0147). Together with the residual diagnostics and independent confirmation, the model is supported for formulation screening and interpolation within the constrained mixture region. Application beyond the investigated component ranges or to different raw-material sources requires additional validation.

XRD, TG, FTIR, and SEM collectively showed continued hydration and progressive microstructural densification with curing age. The C-S-H gel index increased from 2.07% at 3 d to 6.15% at 28 d, while the number of large visible voids decreased and matrix continuity improved. Phase interpretations were based on combined analytical evidence. Durability, long-term volume stability, and life-cycle environmental impacts require further investigation.

## Figures and Tables

**Table 1:** Technical indicators of materials.

Test Item	SS		BFS	
	Measured Value	Technical Specifications	Measured Value	Technical Specifications
Moisture content (%)	0.2	≤1.0	0.1	≤1.0
Density (g/cm^3^)	3.3	≥3.2	2.9	≥2.8
Specific surface area (m^2^/kg)	999	≥350	975	≥400
SO_3_ content (%)	0.2	≤4.0	--	--
Fluidity ratio (%)	--	--	101	≥95
Chloride ion content (%)	0.00	≤0.06	0.01	≤0.06
Loss on ignition (%)	--	--	0.2	≤1.0
Fluidity ratio (%)	104	≥95	--	--

**Table 3:** Formulations and Component Fractions Used in the Constrained Four-Component Mixture Design.

Point	SS (%)	BFS (%)	OPC (%)	DG (%)
A1	38	40	12	10
A2	40	40	10	10
A3	30	46	10	14
A4	30	43	14	13
A5	34	41	15	10
A6	32	43	13	12
A7	30	49	11	10
A8	34	44	10	12
A9	32	47	11	10
A10	38	40	10	12
A11	35	40	13	12
A12	31	40	15	14
A13	35	45	10	10
A14	33	44	13	10
A15	33	43	11	13
A16	34	41	11	14
A17	30	45	15	10

**Table 4:** Mean Strength, Standard Deviation, and Coefficient of Variation of the 7 d Mechanical Test Results.

Mixture	Flexural Strength		Compressive Strength	
	Mean ± SD	CV (%)	Mean ± SD	CV (%)
A1	4.00 ± 0.52	12.99	17.67 ± 1.86	10.54
A2	4.47 ± 0.42	9.32	18.80 ± 1.18	6.26
A3	3.53 ± 0.21	5.89	12.27 ± 0.50	4.08
A4	3.50 ± 0.10	2.86	13.40 ± 0.40	3.02
A5	5.07 ± 0.29	5.70	20.60 ± 0.92	4.48
A6	6.00 ± 0.30	5.00	23.80 ± 0.99	4.14
A7	4.77 ± 1.99	41.66	23.40 ± 1.31	5.59
A8	5.30 ± 0.20	3.77	19.73 ± 1.56	7.90
A9	4.80 ± 0.30	6.25	20.92 ± 1.68	8.03
A10	4.87 ± 0.42	8.55	21.43 ± 1.08	5.03
A11	4.03 ± 2.15	53.43	21.82 ± 1.04	4.78
A12	3.40 ± 0.20	5.88	11.82 ± 0.52	4.39
A13	4.27 ± 0.25	5.90	18.00 ± 0.80	4.46
A14	4.80 ± 0.17	3.61	21.00 ± 0.90	4.30
A15	5.43 ± 0.06	1.06	19.40 ± 0.86	4.42
A16	3.30 ± 0.20	6.06	11.60 ± 0.67	5.80
A17	5.10 ± 0.20	3.92	24.17 ± 1.10	4.55

**Table 6:** Preliminary Raw-Material Cost Estimate for the Selected Composition.

Material	Normalized Mass Fraction	Quantity (kg/t)	Unit Price (CNY/t)	Cost Contribution (CNY/t)
SS	0.3226	322.6	100	32.26
BFS	0.4299	429.9	170	73.08
OPC	0.1313	131.3	275	36.11
DG	0.1162	116.2	40	4.65
Selected binder	1.0000	1000.0	—	146.10
100% OPC reference	1.0000	1000.0	275	275.00

**Table 7:** Semi-Quantitative Indices Derived from the TG Curves.

Curing Age	C-S-H Gels (%)	CH Content (%)	CaCO_3_ Content (%)
3 d	2.07	2.54	2.32
7 d	2.92	1.48	0.57
10 d	3.72	2.03	1.70
28 d	6.15	2.55	1.80

## Data Availability

The original contributions presented in this study are included in the article. Further inquiries can be directed to the corresponding author.
